# Mugharat an-Nachcharini: A specialized sheep-hunting camp reveals high-altitude habitats in the earliest Neolithic of the Central Levant

**DOI:** 10.1371/journal.pone.0227276

**Published:** 2020-01-22

**Authors:** Stephen Rhodes, E. B. Banning, Michael Chazan

**Affiliations:** Department of Anthropology, University of Toronto, Toronto, ON, Canada; University at Buffalo - The State University of New York, UNITED STATES

## Abstract

The earliest Neolithic of southwest Asia is generally perceived and portrayed as a period of emerging economic practices that anticipated full-fledged food-producing economies. This first Neolithic, however, can also be seen as the last gasp of an earlier way of life that remained fundamentally Epipaleolithic in character. While people at this time had begun to cultivate some of the plant foods gathered in preceding periods, and to live for lengthy periods in sites with substantial architecture, they also relied on hunting for a significant portion of their diet and logistical movement across landscapes to exploit diverse environments. The objective of our research on Nachcharini Cave, the only excavated early Neolithic site in the high mountains of northeastern Lebanon, is to evaluate its role in a form of logistical organization not well attested at other sites in the Levant during this period. On the basis of material that Bruce Schroeder excavated in the 1970s, we present here for the first time analyses of faunal and lithic evidence from Nachcharini Cave, along with new radiocarbon dates that place the major occupation layer of the site firmly in the earliest Neolithic. We conclude that Nachcharini was a short-term hunting camp that was periodically used over some two centuries.

## Introduction

The prevailing picture of the shift from mobile hunter-gatherer adaptations to village farming communities in the Middle East portrays a complex process in which changes in settlement, ritual, and mobility preceded clear morphological evidence for subsistence based on domesticated crops and subsequently domesticated animals [1–4]. In this process, the early Pre-Pottery Neolithic A (PPNA), which corresponds to the climatic amelioration that followed the end of the Younger Dryas, was a critical transition during which increased nucleation, more permanent settlement, and examples of monumental architecture provide evidence for social cohesion and display [5–7]. However, while the prevalence of grinding stones signals the important role of plant foods in diet and there is strong evidence for crop cultivation at some sites, evidence for plant domestication is minimal [8–10], and the exploitation of ungulates shows little change from the preceding Natufian period [11].

**Fig 1:**
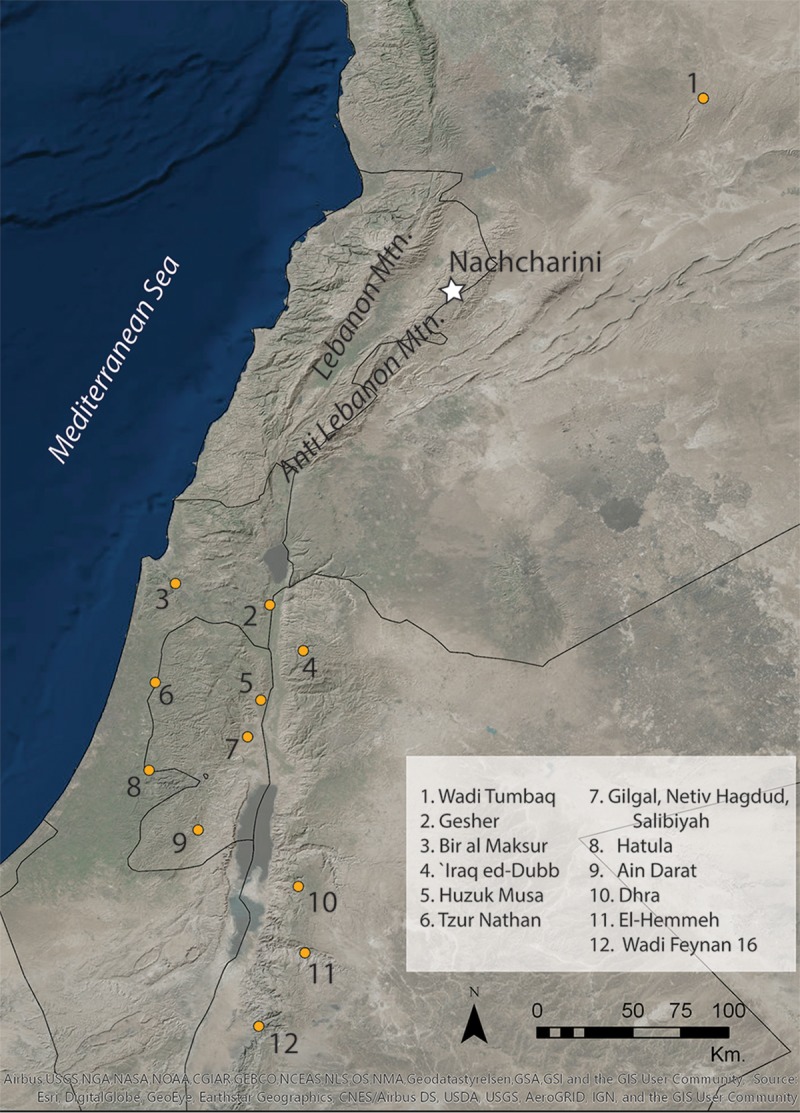
Map of region showing location of Nachcharini Cave and other sites mentioned herein. Map by the authors. Sources: Hillshade: Airbus, USGS, NGA, NASA, NOAA, CGIAR, GEBCO, NLS, OS, NMA, Geodatastyrelsen, GSA, GSI and the GIS User Community; World Imagery (Clarity): Esri, DigitalGlobe, GeoEye, Earthstar Geographics, and the GIS User Community. This work is licensed under the Esri Master License Agreement. Site locations based on researcher database.

We present here the first comprehensive overview of the results of Bruce Schroeder’s excavations at the PPNA site of Mugharat an-Nachcharini in the Anti-Lebanon Mountains (Fig 1), including AMS radiocarbon age determinations, the characteristics of the lithic assemblage, and the composition of the ungulate faunal assemblage. These results confirm Schroeder’s identification of Mugharat an-Nachcharini as a short-term hunting camp, a previously unattested type of PPNA occupation, that was contemporary with the large Sultanian sites of the Jordan Valley and has particularly strong parallels to Netiv Hagdud. Furthermore, Nachcharini is the first PPNA site to show a predominance of sheep in the hunted fauna. These results support the view of PPNA society as consisting of hunter-gatherers who practiced logistical foraging, with both base camps in the form of substantial villages, and specialized sites, such as the hunting camp at Nachcharini.

### Site background

**Fig 2:**
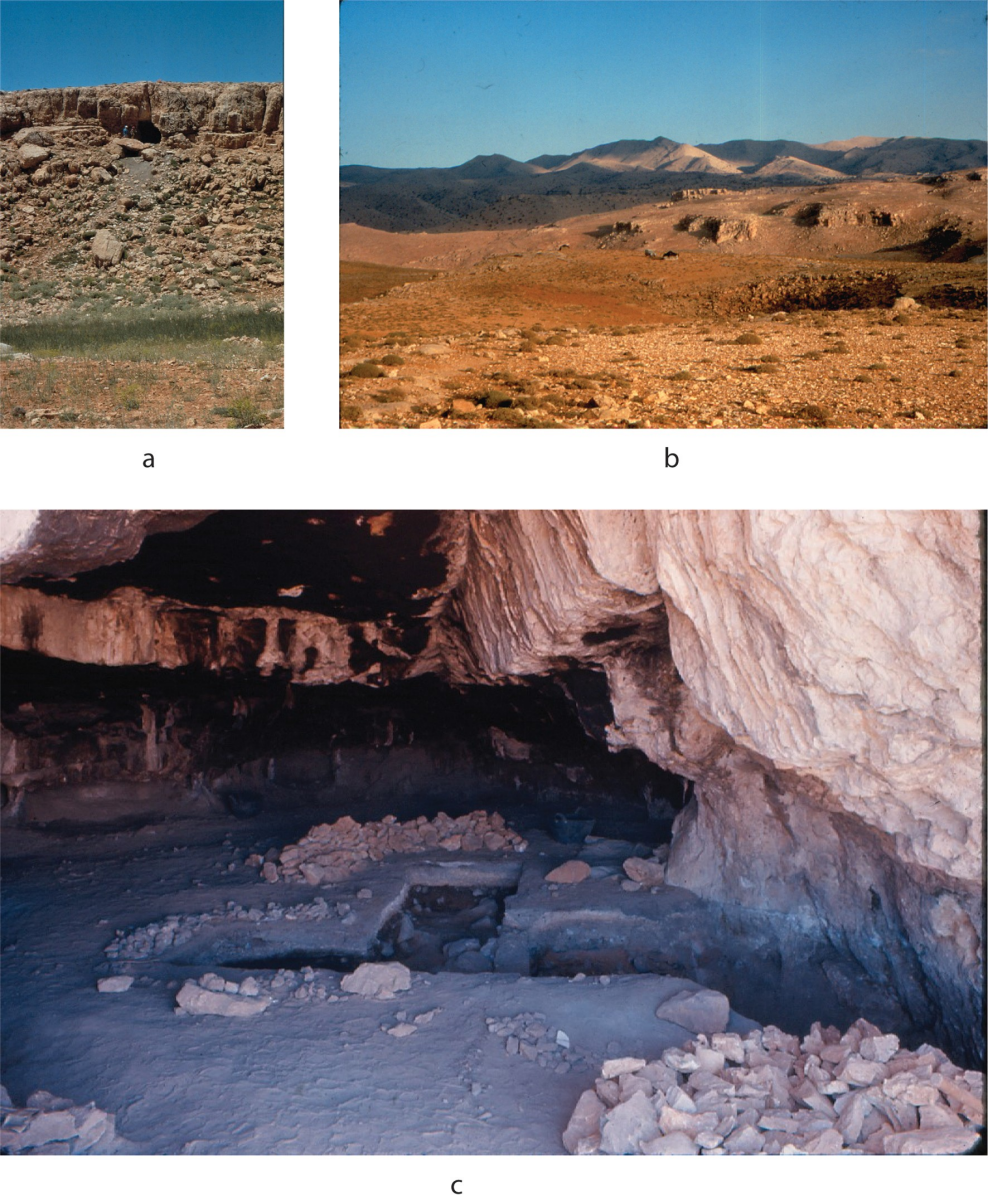
Views of Nachcharini Cave and environs. a) view of cave mouth looking east, b) view of *‘Ard al-Kishek* plateau looking west from cave, c) view of excavation units at end of season, summer 1974, looking east. All photos by B. Schroeder.

Nachcharini Cave (Fig 2) is located at an elevation of ca. 2100 masl in the central *‘Ard al-Kichek* plateau of the Anti-Lebanon Mountains (*al-Jebel ash-Sharqi*) in eastern Lebanon, near the modern border with Syria [12–14]. Unlike the lowland villages that have been the focus of most research on this period, Nachcharini is thus at the highest elevation of any known early Neolithic site in the Levant, making it a truly montane site. Its environs are characterized by rolling land broken by karstic sinkholes that create small, steep-walled canyons and valleys. These landforms provide a good potential habitat for wild sheep, and there are steeper peaks and slopes nearby that would provide habitat for wild goat and mountain gazelle. The presence of deer in the Nachcharini faunal assemblage suggests proximity to wooded areas. During winter, the plateau receives significant snowfall, and snow often persists there until May or June. The plateau may well have sustained vegetation suitable for grazing and browsing herbivores into the late summer, when available resources may have diminished in the adjacent lowlands.

**Fig 3:**
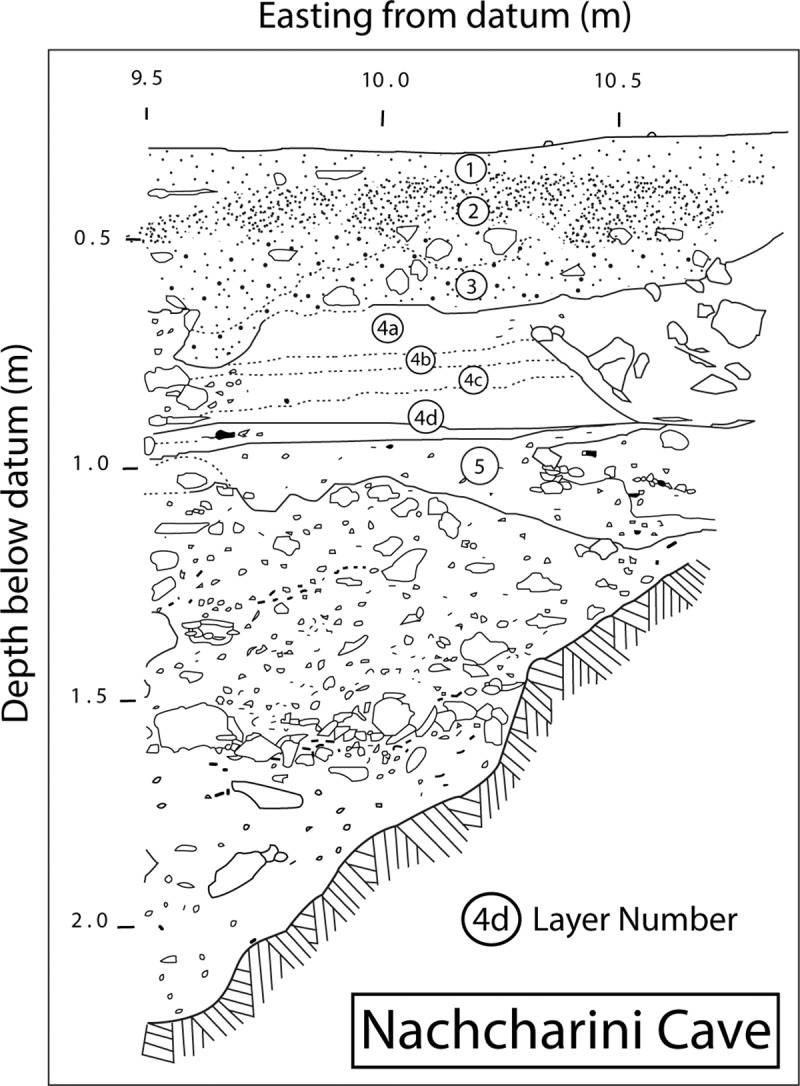
Stratigraphic profile of Schroeder’s excavations at Nachcharini Cave. L. Wadsworth and S. Rhodes after original by B. Schroeder.

**Fig 4:**
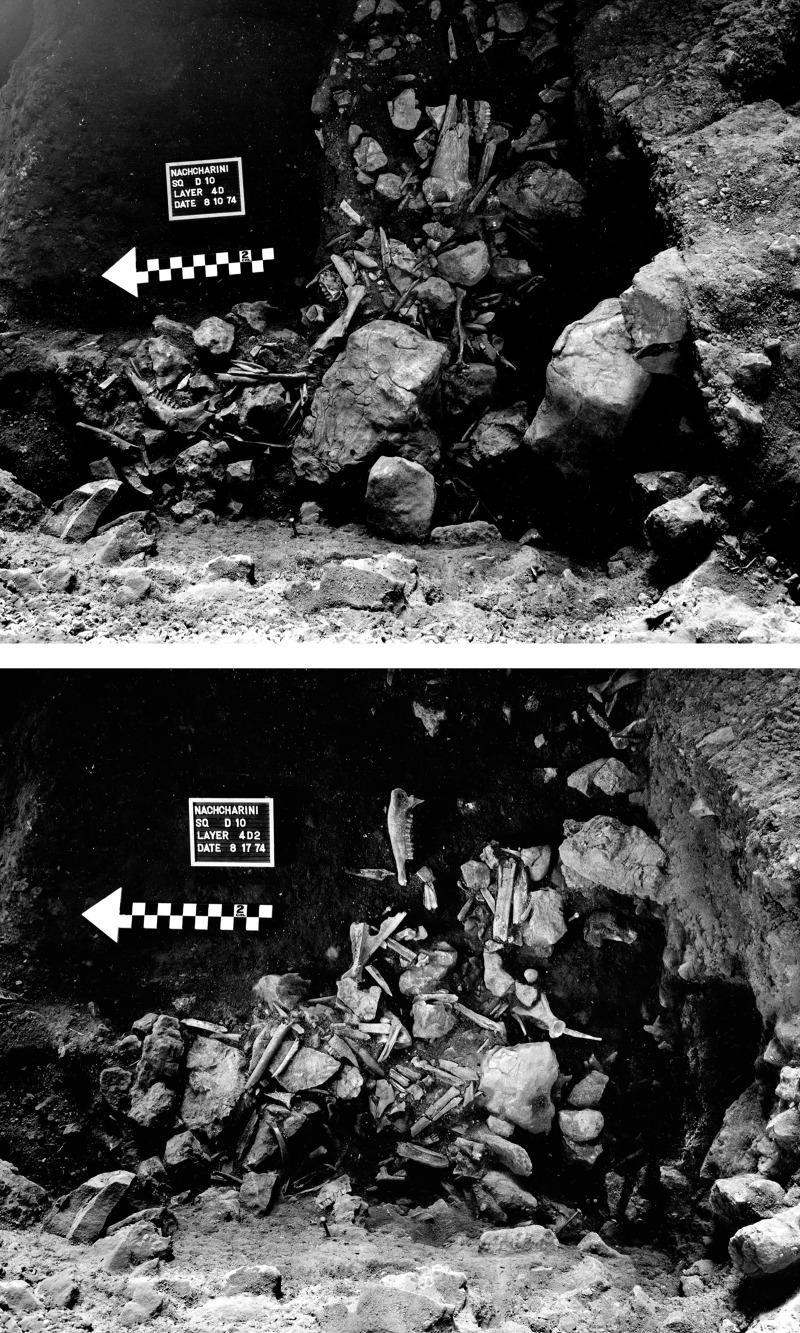
Photographs of layer 4d *in situ* material prior to removal, exemplifying the *décapage* method. Photos by B. Schroeder.

Jacque Besançon discovered Nachcharini Cave about 1970, and Bruce Schroeder subsequently excavated at the site in 1972 and 1974. Significant disturbances at the site substantially reduced the area available for excavation. The 1972 season produced a stratigraphic profile in an exposure of approximately 0.5 m^2^ and pointed to the richness of the PPNA component (Fig 3). The excavation of 1974 expanded the initial exposure of the PPNA to a total of approximately 3 m^2^. Excavation was in 1 m^2^ units, subdivided into 9 subsquares, with baulks left that reduced the excavation area (Fig 2C). Schroeder’s excavation involved attention to full recovery of all materials and is an early example of the use of the *décapage* method, with exposure and photography of *in situ* surfaces of artifacts and fauna preceding any removals. Schroeder identified a sequence of five levels (Fig 3). Here we focus on Layer 4d at the bottom of PPNA Layer 4, in which Schroeder exposed a dense concentration of remains. Layer 4d was reached in three squares (D9, D10, and E9) although part of the area was disturbed by intrusive pits. As Fig 4 shows, Layer 4d was excavated as two surfaces, the upper 4d1 and the immediately underlying 4d2. In 2001, work by the Nachcharini Highland Survey Project confirmed Schroeder’s original stratigraphy and found that looting had further damaged the site [13].

## Materials and methods

All material from Schroeder’s original excavations in 1972 and 1974 was screened through 5mm mesh at the time of excavation, resulting in excellent recovery of faunal remains and lithics. The Department of Anthropology at the University of Toronto curates the collections of material from Nachcharini Cave that Schroeder was permitted to export. The lithics in Toronto constitute diagnostic artifacts from both disturbed contexts and the excavation. A portion of the excavated assemblage was left in Beirut and the current status of this material is unknown. Lithic counts based on the excavation notebooks (see discussion of lithics below) provide an estimate of the total size of the assemblage and make it clear that the Toronto sample lacks non-diagnostic elements that were left in Beirut.

Remarkable preservation of bone collagen in the faunal material has allowed AMS dating on bone, and enables future analysis of ancient DNA from the site. Three samples of bone collagen from square D10, layer 4d in Nachcharini Cave were submitted to the Oxford Radiocarbon Accelerator Unit for AMS radiocarbon dating (samples OxA 27998, OxA 27999, and OxA 28000). These were corrected for fractionation by using measured *δ*^13^C values that ranged from -18.07 to -18.25 (see discussion of radiocarbon dates below). We calibrated and analyzed these dates using the 2013 atmospheric calibration curve in BCal [15–16]. Using a variety of model assumptions, we ran each model at least three times with different random number seeds to check on consistency of results, but the results of only one of these runs appears here.

The faunal material from Nachcharini Cave (layer 4d) is generally well preserved in terms of surface details and overall condition but the specimens are highly fragmented, primarily as a result of intentional perimortem fracture, presumably for marrow extraction. Various post-depositional factors also contributed to fragmentation in some cases. Therefore, the vast majority of specimens cannot be attributed to a specific taxon. Despite these limitations, a significant number of specimens are identifiable to genus or species, providing valuable information about human activities at the site. Based on those ungulate faunal specimens with secure taxonomic attributions, this preliminary faunal analysis reports the Number of Identified Specimens (NISP) for the assemblage, and the Minimum Number of Individuals (MNI) for each taxonomic group. The MNI presented here is based on specimens of right mandibles, which were the most frequently occurring element type in the sample assemblage from Layer 4d. Identification is based on comparisons with the Howard Savage Archaeo-Osteology Collection, University of Toronto, and the Mammalogy Collection, Royal Ontario Museum, as well as established reference material [17–25] Additionally, ages for the most frequent taxonomic groups (sheep/goat, gazelle) were assessed, again on the basis of right mandibles. Determinations of age were based on established criteria [19, 21, 26–29].

## Results

### Radiocarbon dates

**Fig 5:**
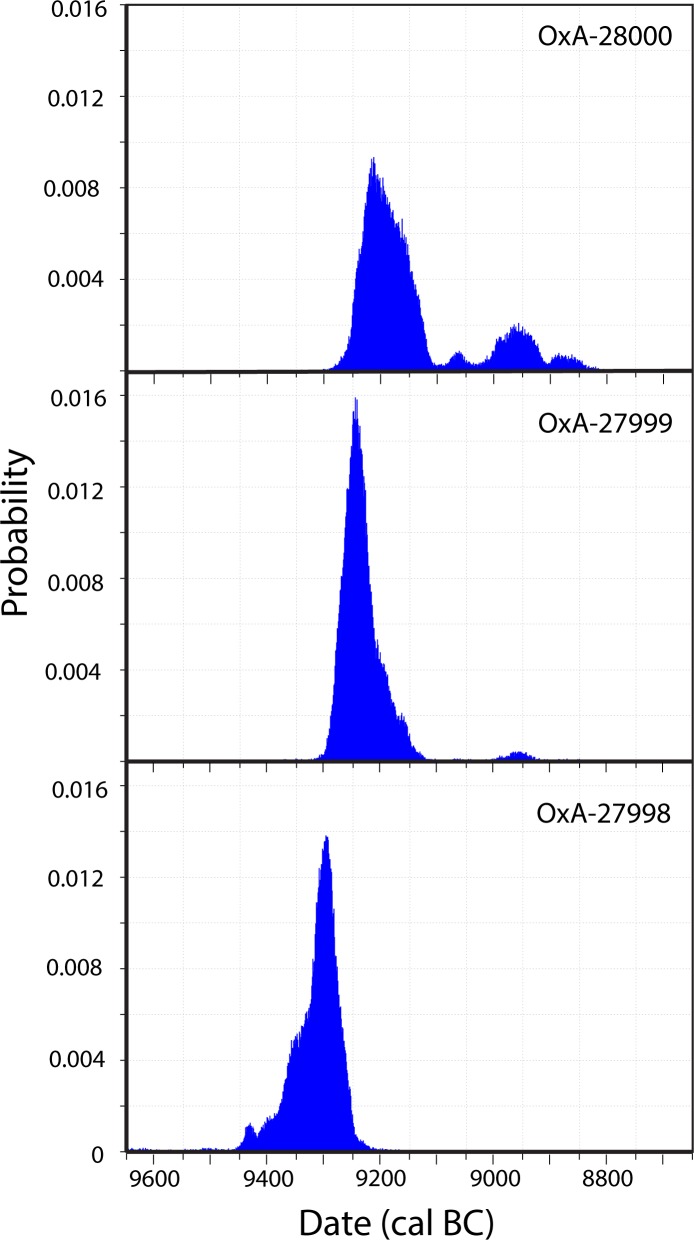
Calibrated probability density functions for samples OxA-27998, OxA-27999, and OxA-28000 from Nachcharini Cave, layer 4d. Calibrated with BCal [15].

Looking first at calibrations without assuming any model for their order or association (an “uninformative” model), OxA-28000 intercepts a plateau in the calibration curve, resulting in a multimodal calibration and relatively long time-span, while OxA-27999 intercepts a steep portion and OxA-27998 falls near the beginning of that portion. OxA-27998 does not overlap very much with the later two, even at 95% confidence, and may pertain to an earlier event (see Table 1 and Fig 5). In this “uninformative” run, the probable elapsed time between OxA-27998 and the next younger date is 17–121 years at 68% and -25 to 233 years at 95% credible interval. The probability that OxA-27998 is from an earlier event than OxA-27999 is 0.96.

**Table 1:** Radiocarbon determinations from square D10, layer 4d at Nachcharini. With calibrated credible intervals (cal BC) for “uninformative” calibrations (top), with two of the determinations grouped (middle), and with those two pooled (bottom). Note: Calibrated dates are from one of three runs, each with a different random-number seed, to check on consistency. All gave closely similar results.

Lab Number	Context	δ^13^C	Determination	95% CI	68% CI
OxA-27998	D10-218	-18.25	9875 ± 45	9416–9249	9342–9272
OxA-27999	D10-4d2-F26	-18.20	9745 ± 45	9297–9149	9276–9217
OxA-28000	D10-313-F343	-18.07	9665 ± 45	9268–9113 9070–9057 9009–8916	9246–9146
With OxA-27999 and 28000 grouped:					
OxA-27998				9455–9248	9362–9280
OxA-27999				9298–9136 8972–8941	9275–9211
OxA-28000				9261–9116 9076–9053 9011–8913 8901–8848	9246–9131
With OxA-27999 and OxA-28000 pooled					
OxA-27998				9452–9247	9360–9279
Pooled Event				9267–9137 8974–8940	9249–9185

Grouping the later two determinations and comparing them with the first, with no assumptions about their relationship, widens the gap somewhat (elapsed time between OxA-27998 and OxA-27999 of 26–151 years at 68% credible interval), likely showing sensitivity to the model assumption. Assuming that OxA-27999 and OxA-28000 pertain to the same (pooled) event, and OxA-27998 a different one predictably results in an even longer estimate of elapsed time between the older event and the younger one, 48 to 172 years at 68%. However, a test of the hypothesis that the older date falls within the interval of the two younger dates yields a probability of 0.908, despite the fact that the model assumptions have exaggerated the difference between the older date and the other two.

Whether the dates and their associated faunal remains pertain to a single event around 9200 cal BC or to a series of events over a century or two, they generally fall well within the range of PPNA sites with which they share some other aspects of material culture. At Netiv Hagdud, for example, and using the same methods as for the Nachcharini dates, the beginning of occupation appears to be ca. 9480–9155 cal BC and its end around 9255–8800 cal BC (95% credible intervals from published data) [30]. A probability analysis indicates that the probability that each of the Nachcharini samples falls within this range is 0.306, 0.654, and 0.58, while the actual dates from Netiv Hagdud range from 9411–8940 (RT-762C, 9970 ± 150) to 9280–8928 cal BC (Pta-4556, 9660 ± 70, both at 95% CI), not very different from the earliest and latest dates from Nachcharini. The cave site of ‘Iraq ad-Dubb, another exception to the more familiar lowland sites, has dates from its PPNA levels of 9231–8802, 9741–9266 and 9813–9245 cal BC (95% CI, AA-38140, 9592 ± 64, AA-38145, 9941 ± 72, and OxA-2567, 9950 ± 100) [31], with greater spread but over much the same period as Nachcharini.

### Lithics

The collection in Toronto does not include the complete excavated lithic assemblage, consisting almost exclusively of formal retouched tools. We cannot be certain that all formal tools recovered are present, although the collection matches well with the counts of retouched tools found in the excavation records. Data in the excavation records make it clear that unretouched debitage and cores did not occur in the high densities that one would expect if a complete production sequence took place on site (Table 2). The low frequency of retouched flakes (including formal tool types such as endscrapers) and absence of bifacially worked pieces further emphasizes the specialized nature of the Nachcharini assemblage in the context of the PPNA [32–34].

**Table 2:** Total count of lithics by excavation square from Layer 4d, Nachcharini Cave based on field notebooks.

Excavation Square	# of Lithics
D10	80
D9	132
E9	33
**Total**	**245**

**Fig 6:**
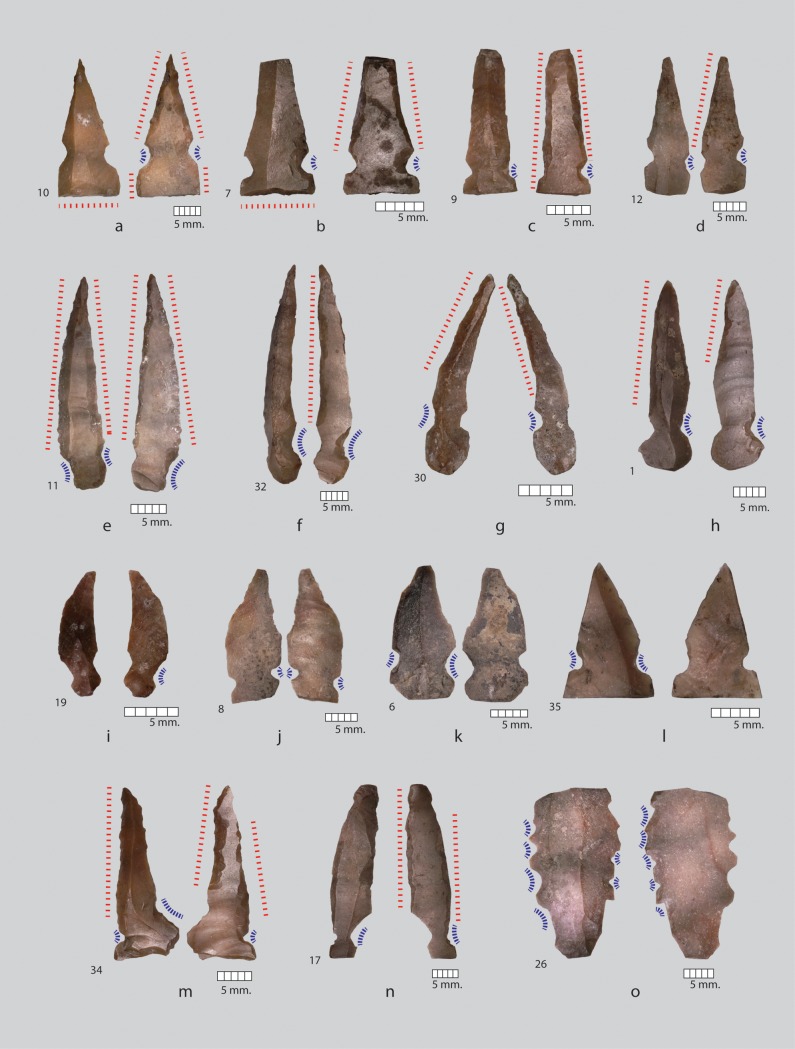
El Khiam points and variants from Nachcharini, St. 4d. Red dotted lines indicate linear retouch on an edge, blue dashed lines indicate notching. Number is the artifact designation for analysis. Side based on dorsal view. a: Distal blade with abrupt dorsal truncation at proximal end, ventral right and left marginal retouch, and ventral notching on left and right. b: Distal blade fragment with impact fracture at distal end, abrupt dorsal truncation at proximal end, ventral right and left marginal retouch above notches, dorsal notch on left margin and ventral notch on right margins. c: Distal blade fragment with impact fracture at distal end, ventral marginal retouch on right margin, ventral marginal retouch above notch on left margin, dorsal notch on left margin, ventral notch on right margin. d. Distal blade fragment with impact fracture at distal end, ventral marginal retouch on right margin above notch, dorsal notch on left margin, ventral notch on right margin. e. Blade with twisted profile, bifacial retouch on both margins, bifacial notch on left margin, dorsal notch on right margin, notches flush with base. f. Twisted blade with dorsal notch on right margin, ventral notch on left margin, ventral retouch above notch on right margin. g. Twisted bladelet with dorsal notch on left margin, ventral notch on right margin, dorsal left marginal retouch, and ventral right marginal retouch. Note offset of point from long axis. h. Blade with left dorsal margin retouch, right margin ventral retouch restricted to the upper part of the blade, dorsal notch on right side, ventral notch on left side. i. Twisted bladelet with ventral notch on left margin, notch on left margin appears to be the unretouched morphology of the bladelet. j. Distal bladelet fragment with bifacial notch on right margin, ventral notch on left margin. k Bladelet with dorsal notches on left and right margins. l. Distal blade fragment with dorsal notch on both sides. m. Irregular distal blade fragment with bifacial notch on left margin, dorsal notch on right margin, bifacial marginal retouch above notch on left margin, and ventral retouch above notch on right margin. n. Blade with dorsal notch on right distal end and ventral notch on left distal end, ventral retouch above notch on both margins. The tip of this piece is not a point and the orientation is unusual with the notches at the distal end of the bladelet. o. Blade fragment with a series of four dorsal notches on left margin and two bifacial notches between two ventral notches on right margin.

**Fig 7:**
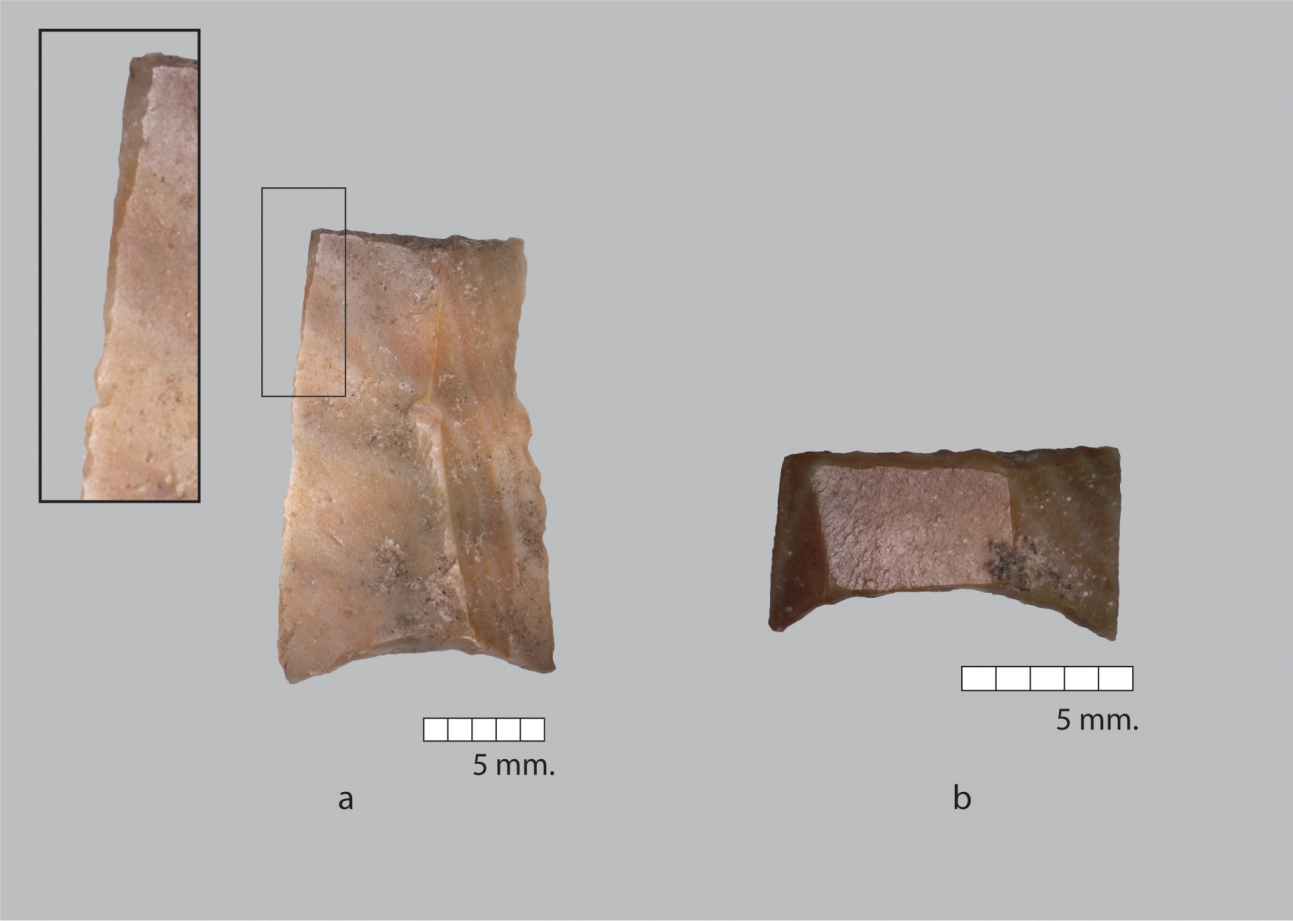
Hagdud truncations on blade fragments with concave base and straight distal end. Inset shows edge damage on upper left margin of a.

The dominant tool types in the Nachcharini assemblage from layer 4d are Khiam points and Hagdud truncations (Table 3, Figs 6 and 7). Hagdud truncations are defined as having “two parallel retouched truncations, proximally and distally across the width of a blade or a bladelet fragment, while two lateral edges (remnants of the original blade/bladelet edges), were not retouched”[35]. Khiam points are described as points “with one pair (or more) of notches near the base or along the sides. The tip is retouched and the base is usually truncated”[36]. Four pieces were identified as retouched point tip fragments. At Nachcharini, there is variability in the pattern of retouch, with basal truncations rare (Fig 6A and 6B) and marginal retouch sometimes absent (Fig 6I–6L). Marginal retouch can be dorsal (Fig 6D), ventral (Fig 6A–6C, 6F and 6N), alternate (dorsal on one side and ventral on the other, Fig 6G and 6H), or bifacial (Fig 6E and 6M). One piece has a series of notches on each margin (Fig 6O).

**Table 3:** Typological breakdown of Layer 4d artifacts in the Toronto lithic sample from Nachcharini Cave.

Tool Type	Number	Percent
Khiam Points	14	20.6
Hagdud Truncations	12	17.6
Salabiya Points	1	1.5
Misc. Points	1	1.5
Awls	9	13.2
Misc. Retouched	24	35.3
Unretouched	7	10.3
**Total**	**68**	**100**

**Fig 8:**
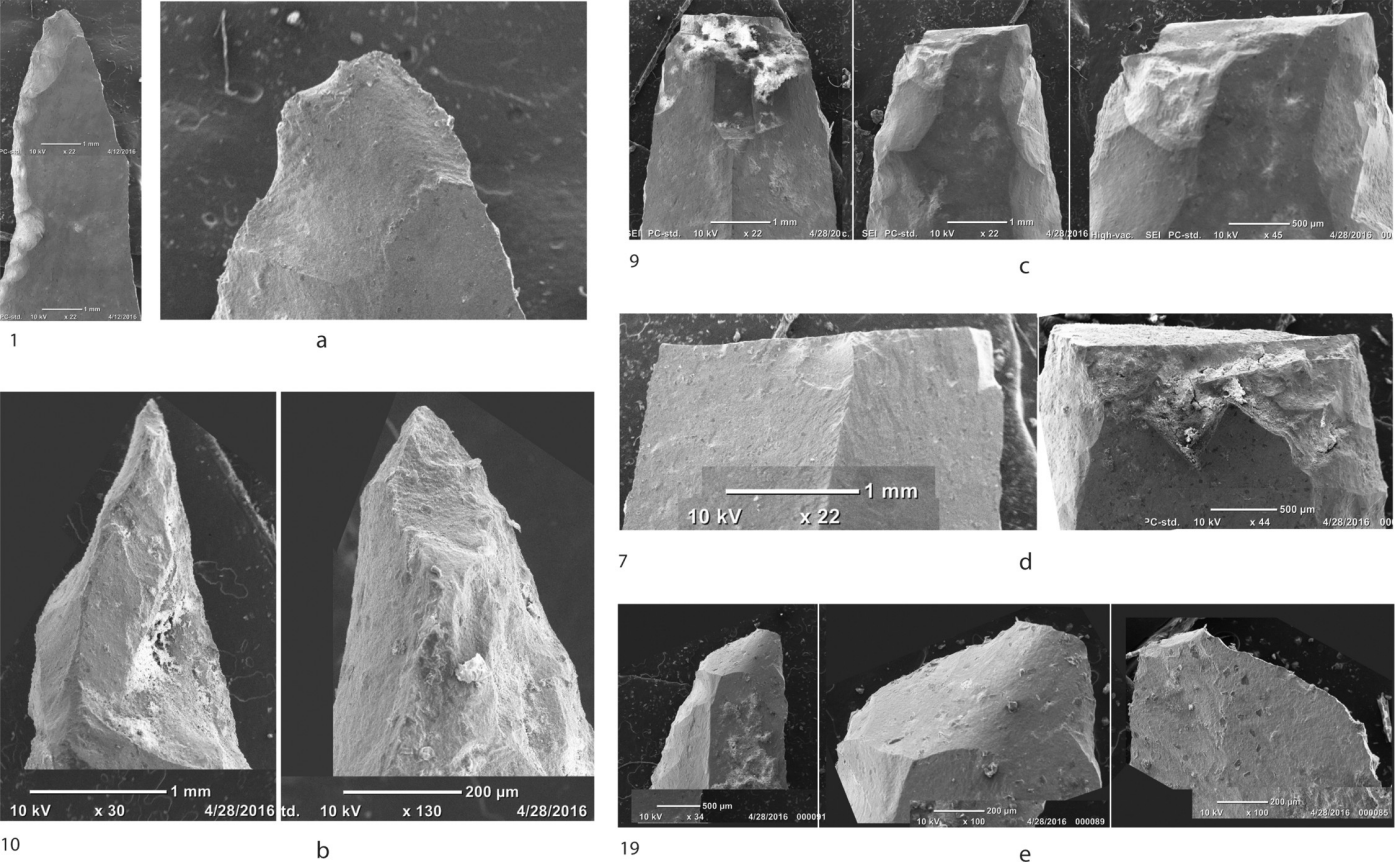
Scanning electron microscope images of damage to tips of El Khiam points from Nachcharini. A. SPL 1 (Fig 6H). Left, overview showing ventral marginal retouch. Right, detail ventral face. Impact fracture, intermediate between bending and cone initiation, initiate from left ventral margin, feather termination at right ventral margin. B. SPL 10 (Fig 6A). Left, overview showing retouch on ventral face. Right, detail of ventral face. Impact fracture, unclear initiation from the tip off the ventral surface with intermediate step/hinge termination. C. SPL 9 (Fig 6C). Left, overview dorsal face. Right, overview ventral face. Center, detail ventral face. Break at distal end with a series of stepped spin-off fractures of dorsal face. D. SPL 7 (Fig 6B). Left, overview dorsal face. Right, overview ventral face. Break at distal end with a series of stepped spin off fractures off ventral face, burin fracture off right margin, and feathered spin off fracture off dorsal face. E. SPL 19 (Fig 6I). Left, overview of dorsal face showing marginal retouch. Center, detail of dorsal face. Right, detail of ventral face. Bending fracture on dorsal face initiated on left margin with feather termination on right margin. Spin off fracture (?) on tip on ventral face. Terminology based on Coppe and Rots 2017 [41].

The association of Khiam points and Hagdud truncations at Nachcharini supports the argument that these pieces were elements in composite hunting tools [37–38]. Impact fractures are very rare on the Hadgud truncations but are clearly apparent on the distal end of many of the Khiam points. Examination of the points with distal fracture shows massive failure consistent with high-velocity impact and there is little doubt that these are armatures for light projectiles (Fig 8). The small size of the Khiam points is somewhat surprising, although small points can still have considerable lethal potential [38–40]. Technologically, the Khiam points range from pieces made on flat blades (Fig 6A, 6B and 6L) to retouched, thin, twisted blades (Fig 6E–6H) and small flakes (Fig 6I–6K) that demonstrate a high degree of expediency in blank production for a formal tool type. Some of the blanks are remarkably irregular, such as the asymmetrical blade in Fig 6M and the piece shown in Fig 6N, where the notches are actually on the distal end of an irregular blade and the tip (proximal end) does not actually reach a point. There is a sense that, when the hunters at Nachcharini were retooling points, they frequently recycled broken pieces or made use of unstandardized debitage. However, this trait is not shared with the Hagdud truncations, which are consistently on blade fragments.

**Fig 9:**
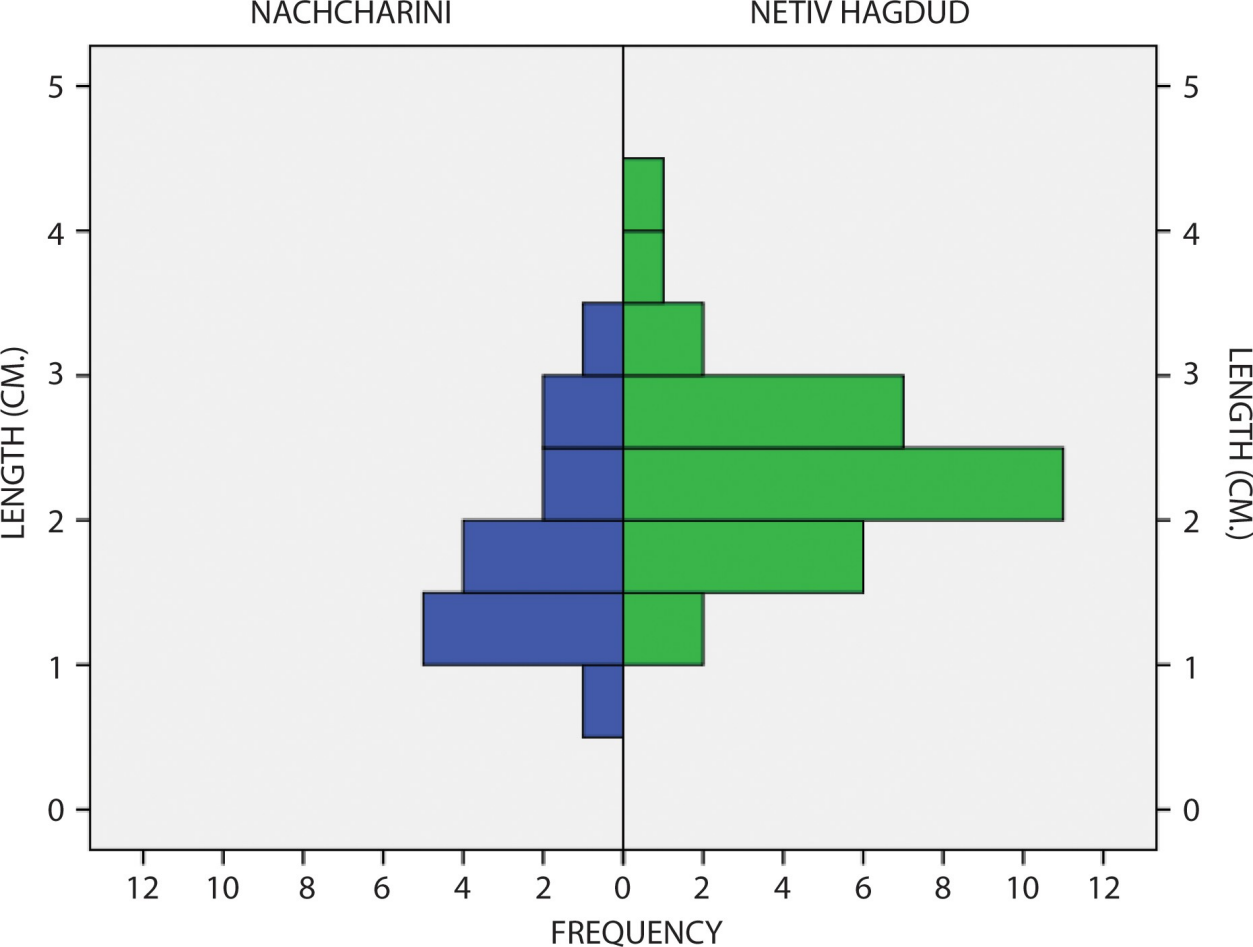
Comparison of size distribution of points from Nachcharini and Netiv Hagdud.

Regionally, there is high degree of inter-site variability in the ratio of points to Hagdud truncations. Although sample size is an issue, Nachcharini and Netiv Hagdud nonetheless appear to be the only sites with a balance between the number of points and Hagdud truncations (see S1 File). While these tools might have been components of composite points [37], the inter-site variability suggests that this may not have been universal. At Dhra‘ and Wadi Feinan 16, for example, there is evidence for use of Khiam points as perforators, a function that is not apparent at Nachcharini, and accordingly these two sites have a high ratio of Khiam points to Hagdud truncations [42]. The most complete data for metrics of Khiam points comes from Netiv Hagdud [30], a site that is, as discussed above, contemporary with Nachcharini. At Netiv Hagdud, the Khiam points are generally longer than at Nachcharini, although there is overlap in size (Fig 9). However, the Netiv Hagdud assemblage also includes very small points and pieces that show the kind of expediency found at Nachcharini.

### Fauna

**Fig 10:**
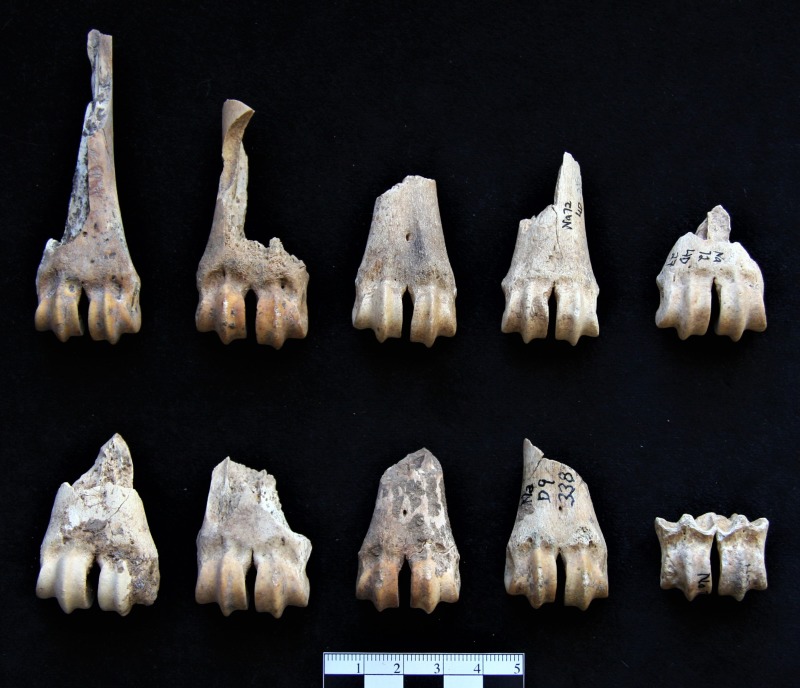
Distal metapodia of specimens attributed to *Ovis cf*. *orientalis* from Nachcharini Cave, layer 4d.

Table 4 shows the frequency of faunal remains of different taxa from Nachcharini layer 4d. Wild sheep (*Ovis cf*. *orientalis*) are the most represented taxon with at least seven individuals present, while Mountain gazelle (*G*. *gazella*) are represented by a minimum of five individuals. The undetermined *Ovis/Capra* category also has an MNI of five, and of these, four are identified as “probable *Ovis*,” and one as “probable *Capra*.” *Capra* (*C*. *aegagrus*, possibly *C*. *ibex*) itself has an MNI of two. So, sheep and goat together represent a minimum of 14 individual animals, while gazelle represent five, and deer (*Cervus elaphus* and possibly *Dama mesopotamica*,) just two. Again, MNI for *Ovis*, *Capra*, *and Gazella* here is calculated on the basis of right mandibles. Age information shows that the primary focus of hunting at PPNA Nachcharini was on adults of prime age, although several sub-adult animals are also present in the Layer 4d sample. Fig 10 shows examples of distal metapodia attributed to wild sheep (*Ovis cf*. *orientalis*) from Nachcharini Cave layer 4d.

**Table 4:** Representation of ungulate fauna from Nachcharini Cave layer 4d (PPNA).

Taxon	Common Name	MNI	NISP	%NISP
*Ovis sp*.	Wild sheep	7	64	18.0%
*Capra spp*.	Wild goat	2	16	4.5%
*Ovis/Capra*	Wild sheep or goat	5	204	57.5%
*Gazella gazella*	Mountain gazelle	5	50	14.1%
*Cervus elaphus*	Red deer	1	8	2.3%
Cervidae indet.	Deer (indet.)	1	13	3.7%
**Totals**	** **	**21**	**355**	**100.0%**

Some differences in post-cranial element representation were apparent. The sheep/goat and gazelle bones show fairly complete representation of all skeletal regions (head, axial skeleton, limbs, feet), suggesting that primary butchery of these animals occurred within the cave. The deer bones show a different pattern, with a more uneven representation of skeletal regions and more highly fragmented specimens, perhaps suggesting offsite butchery or accumulation by scavengers. Again, these conclusions are tentative due to small sample size and various taphonomic factors, mentioned above. A comprehensive report on the taphonomy and fauna from all excavated units at the site is forthcoming (Rhodes, in prep.).

The fauna from Nachcharini layer 4d shows a distinct focus on ungulates, with little evidence of the “broad spectrum” of animal resources seen at many contemporaneous Levantine sites (e.g., Netiv Hagdud) [43]. Within the ungulate assemblage, the caprinae (sheep and goat) strongly predominate over other prey, such as gazelle and deer. Furthermore, among the caprinae, there is a much higher incidence of sheep than of goat, making this site unique for this region and time period. This affords the possibility, however remote, that the PPNA hunters who created the assemblage could have been engaged in some sort of incipient herd management [44–45] of caprines prior to the unambiguous appearance of caprine domestication in the region during the subsequent PPNB [46]. Unfortunately, small sample sizes and taphonomic factors obscure the picture at Nachcharini too much for a thorough evaluation of this hypothesis, and future aDNA studies may shed more light on this question. At the least, the confirmed presence of wild sheep in the Anti-Lebanon Mountains, and in adjacent areas to the east [47], identifies a possible ancestral source for domesticated sheep in the mountains of the Central Levant, as well as further southeast near Jebel Druze [48].

## Discussion

The faunal and lithic data from Schroeder’s excavation at Nachcharini Cave present a vivid picture of a task-specific site focused on the hunting of ungulates, especially sheep. The radiocarbon dates firmly place this occupation within the PPNA, nearly synchronous with the occupation of Netiv Hagdud in the Jordan Valley. Lithic data, including the ratio of Khiam points to Hagdud truncations, also indicates a strong typological similarity between these two sites in these two tool types and probably their use in projectile armatures. However, from a broader perspective, Netiv Hagdud’s lithic assemblage exhibits a diversity not found at Nachcharini. The Khiam points from Nachcharini have a high degree of expediency that is consistent with retooling and there is no evidence for primary core reduction at the site. It appears that the PPNA hunters at Nachcharini were adept at using available materials to retool their hunting projectiles as needed. The dense accumulation of fauna in layer 4d at Nachcharini is consistent with intensive butchery, although it will require further taphonomic analysis to elucidate the nature of faunal exploitation. The three radiocarbon dates from the site are reasonably close but leave open the possibility that Stratum 4d is a palimpsest of multiple site visits over a period of up to two centuries.

Nachcharini provides clear evidence that PPNA mobility included task-specific groups that moved seasonally to exploit resources well outside the vicinity of large sites with architecture. The fauna from Nachcharini layer 4d shows a distinct focus on ungulates, with little evidence of exploitation of the “broad spectrum” of animal resources seen at many contemporaneous Levantine sites, and a strong predominance of caprinae over other ungulates. Furthermore, these unusually exhibit more sheep than goat, affording the possibility of incipient herd management at this early date [44–45]. Nachcharini’s layer 4d fits well with Binford’s conception of a foraging camp, which presumably existed within a larger framework of logistical mobility [49–50]. Copeland suggested such a possibility, but also pointed out that Nachcharini Cave may simply have served as a convenient waypoint for groups crossing the Anti-Lebanon Mountains between the Beqaa’ Valley in Lebanon and al-Majjar depression in Syria [12]. However, this latter suggestion does not account for the density of fauna, the restricted tool assemblage, or the typological similarity of Nachcharini to the Jordan Valley sites.

The reanalysis of material from Schroeder’s excavation at Nachcharini demonstrates not only the importance of this site but also some of the limitations of our understanding of PPNA society. The focus of research on large village sites, which, on the basis of evidence from Nachcharini, can be understood as base camps within a residential mobility strategy, probably provides only a partial perspective. Sites such as Iraq ad-Dubb, which is perched near the top of a cliff with limited space for occupation yet still has architectural remains dating to the PPNA [31], likely represents another type of settlement that was part of a PPNA mobility system [31, 51]. Research on the PPNA that has focused almost exclusively on the Jordan Valley leaves many gaps. For our understanding of Nachcharini, the absence of information for the PPNA in the Amuq and Beqaa‘ Valleys is particularly troubling [52, 53]. There is room to ask whether the PPNA included sites, similar to Late Natufian Hilazon Tachtit, that served as a focus for feasting events that brought different communities together. From this perspective, the monumental architecture at Gobekli Tepe [54, 55], toward the northern end of the PPNA world, might be understood within the context of a mobile hunter gatherer adaptation.

## Conclusion

The PPNA was a dynamic period with diverse adaptations that appear to have included plant cultivation and perhaps incipient herd management. This period also saw radical developments in site structure and monumental architecture in some locations. PPNA groups voyaged widely, as indicated by expansion into Cyprus [56–58] and recent discovery of a PPNA site in Saudi Arabia [59]. The evidence from Nachcharini exemplifies the use of diverse resource areas such as montane highlands, highlighting the diversity of subsistence activities in the PPNA. There is a risk of distorting our understanding of PPNA societies by trying to fit them into a binary opposition between hunter-gatherers and village farmers [1]. Nachcharini provides a vivid indication of the role of hunting during the PPNA, not only as a facet of activity on large sites with a diversified economy, but also in seasonal exploitation of particular resources far removed from those large sites.

## Supporting information

S1 File Nachcharini 4d lithics specimen numbers and additional data (NA lithics). (XLSX)

S2 File Nachcharini 4d faunal specimen numbers list (Nach faunal specimen numbers). (XLSX)
